# Varicella-zoster virus presenting with nasal tip necrosis

**DOI:** 10.1016/j.jdcr.2024.11.016

**Published:** 2024-11-26

**Authors:** Mariah Estill, Jesse Kasim, Barbara Wilson, Keely Marshall, Karolyn A. Wanat

**Affiliations:** a Department of Dermatology, Medical College of Wisconsin, Milwaukee, Wisconsin; b School of Medicine, Georgetown University, Washington, District of Columbia

**Keywords:** nasal tip necrosis, Varicella-zoster virus

## Introduction

Varicella-zoster virus (VZV) is a human herpes virus responsible for varicella (chicken pox) and zoster (shingles). Although VZV infection classically presents on the skin as grouped vesicles on a red base, the cutaneous clinical manifestations of VZV can be diverse and include necrosis of the nasal tip. We report 2 cases of VZV infection presenting as acute onset progressive nontender nasal tip necrosis.

## Case reports

### Patient 1

**Fig 1:**
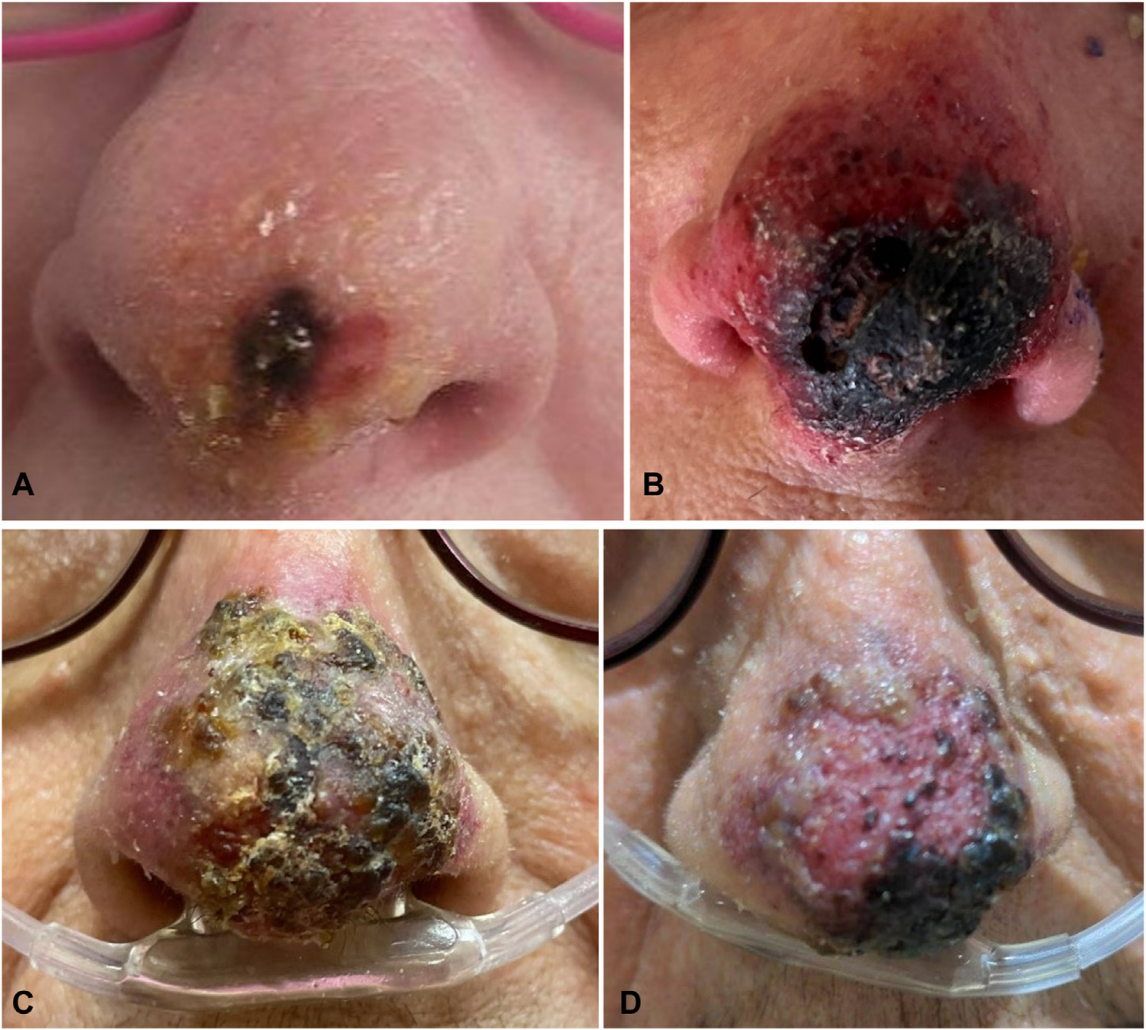
Clinical images of nasal tip necrosis in patients 1 and 2. Patient 1 day 2 (**A**) and day 7 (**B**); Patient 2 day 10 (**C**) and day 14 (**D**).

A 68-year-old woman presented with a 6-day history of an acute onset ulcerating nontender plaque on the nasal tip. Initially starting as 2 clear fluid-filled blisters, the nasal lesions rapidly increased over a few days, turning black (Fig 1, *A*). The patient was initially seen at an outside institution and was treated with amphotericin, vancomycin, and cefepime without improvement. At presentation to our institution, on physical exam, there was a painless verrucous, hemorrhagic crusted, plaque on the distal nasal tip extending toward the bilateral ala (Fig 1, *B*). A day later, multiple pink papules and pustules developed scattered on the forehead and back bilaterally without favoring a dermatome. The patient had a history of parotid cancer status postresection, but no recent treatment with chemotherapy and was otherwise healthy without known immunosuppression (Table I).

**Table I:** Presentation characteristics of the 2 reported cases

	Patient 1	Patient 2
Age (in y)	68	77
Duration of symptoms	Six days	Two weeks
Systemic symptoms	None	None
Pertinent past medical history	Parotid cancer s/p resection	Seborrheic dermatitis
	Normal pressure hydrocephalus	Sebaceous hyperplasia
	Epilepsy	Carcinoid tumor in lung
	Depression	Ductal carcinoma in situ
		Chronic kidney disease
		Liver cirrhosis and failure
		Recurrent urinary tract infections
		Pneumonia
		acute C-spine compression
Previous VZV exposures	Chickenpox as child, no known vaccinations	Unknown prior exposure, no known vaccinations
Previous treatments	Amphotericin, vancomycin, cefepime	None

**Fig 2:**
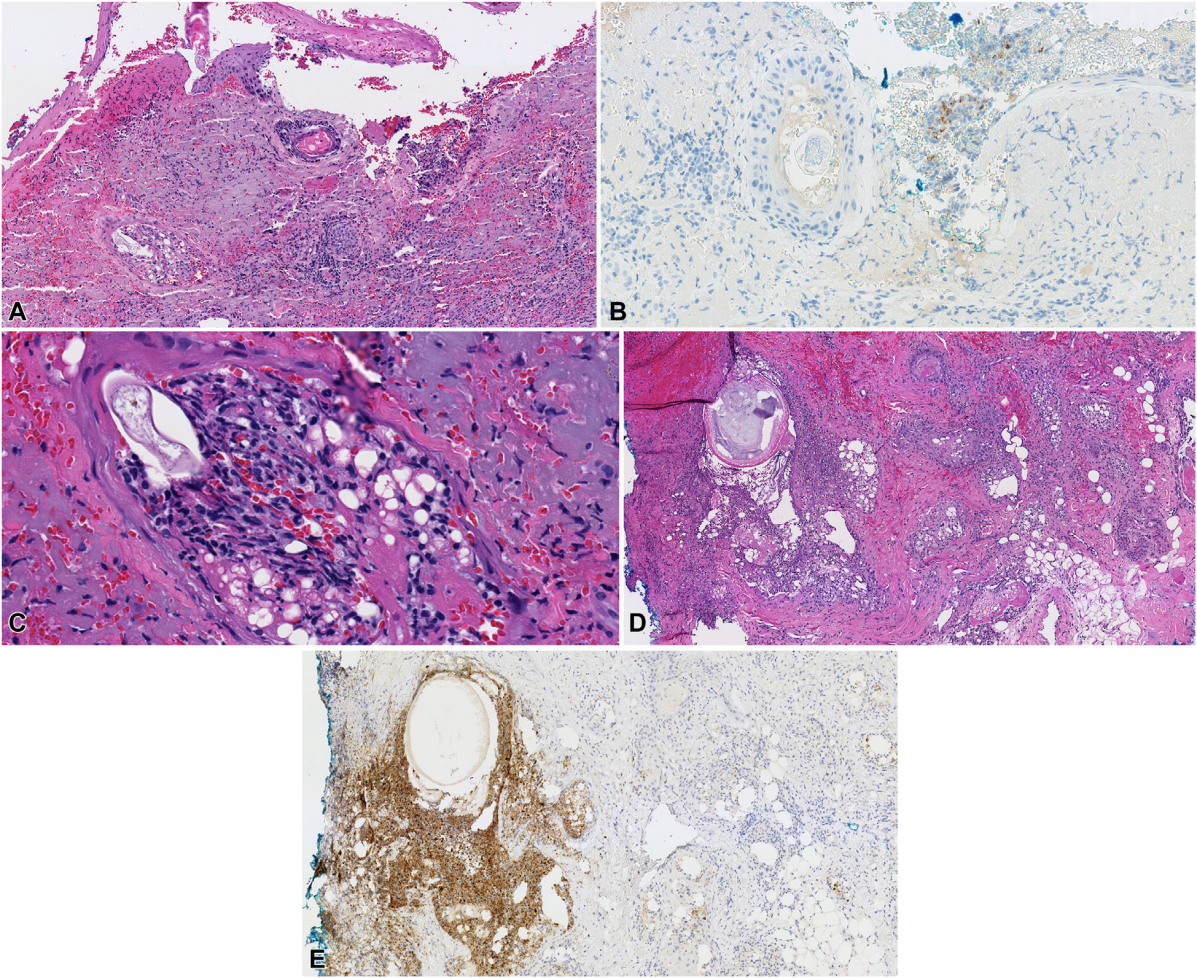
Histopathologic findings of the 2 reported cases. **A,** Punch biopsy of nose in patient 1 demonstrates ulceration with underlying sebaceous gland necrosis and brisk acute inflammation (20×, hematoxylin-eosin). **B,** VZV immunohistochemical staining was positive (20×). **C,** Demodex mite (40×, hematoxylin-eosin). **D,** Punch biopsy of nasal tip in patient 2 demonstrates necrosis of follicles and sebaceous glands (20×, hematoxylin-eosin). **E,** VZV immunostaining positive (20×).

Biopsies of the nose demonstrated ulceration with underlying sebaceous gland necrosis, brisk acute inflammation (Fig 2, *A*) and multiple demodex mites (Fig 2, *C*). Gram and periodic acid-Schiff were negative. herpes simplex virus immunostain and polymerase chain reaction (PCR) was negative. VZV PCR from nasal tip and VZV immunohistochemical stain of biopsy was positive (Fig 2, *B*).

Patient was diagnosed with a disseminated VZV infection after VZV PCR was positive from forehead and other lesions were noted on her back. The patient denied any ocular symptoms but she had a headache and developed conjunctival redness. Ophthalmology consultation found vesicles on lower eye lid but no keratitis or other concerns for ocular involvement of VZV.

Additional lab work-up was remarkable for pancytopenia (Hb 10.5 g/dL, white blood count 3700/uL, platelet 81,000/uL) and a monoclonal gammopathy with low level biclonal IgG kappa and free lambda present on electrophoresis. Kappa and lambda light chains are both elevated (free kappa 107 mg/L, free lambda 56.6 mg/L) with a slightly elevated ratio (1.89).

The patient reported a history of chickenpox as a child and no known vaccination. The patient was treated with intravenous acyclovir 10 mg/kg intravenous q12 hours for 9 days and transitioned to oral valacyclovir 1000 mg twice daily for 5 days at discharge with improvement in symptoms. Outpatient follow up with hematology was recommended for further evaluation of low blood counts or immunodeficiency as a possible underlying cause of disseminated VZV infection, but the patient was lost to follow up.

### Patient 2

A 77-year-old woman presented to her outpatient dermatologist with a 2-week history of nontender acute onset progressive nasal tip necrosis (Fig 1, *C*). The patient had a history of seborrheic dermatitis, sebaceous hyperplasia, and in the preceding months had numerous hospitalizations for acute C-spine compression and anasarca in the setting of liver failure, recurrent urinary tract infection, and pneumonia. On examination, there was a pink plaque with hemorrhagic crust on the nasal tip with no other areas of involvement (Fig 1, *D*). It was unknown if there was any trauma to the nose, but the patient reported the lesion seemed to start as a bruise. A biopsy performed from the nasal tip showed prominent folliculosebaceous gland necrosis with acute inflammation (Fig 2, *D*). periodic acid-Schiff, AFB, and herpes simplex virus 1/2 stains were negative. VZV PCR was not collected, but VZV immunohistochemical stain of the biopsy was positive (Fig 2, *E*).

The patient had no known prior varicella exposure or history of vaccination. There were no ocular symptoms or findings on exam, and no referral was made to ophthalmology. She was treated with oral valacyclovir 1000 mg three times daily and topical mupirocin 2% daily for 1 week. At follow up 6 months later, her nose healed completely without scarring or any complications.

## Discussion

Rapidly progressive necrosis of the nose poses a significant risk of disfigurement and functional impairment in patients. Nasal tip necrosis is a rare presentation of VZV with only 2 previously reported cases in the literature in patients with underlying hypogammaglobulinemia and immunosuppression in the setting of systemic lupus.^1^^,^^2^ The differential diagnosis for nasal tip necrosis is broad and includes infections such as viruses (herpes simplex virus, VZV, or Mpox), fungi (blastomycosis, histoplasmosis, or angioinvasive infections such as mucormycosis), and bacteria. Noninfectious causes include malignancy and vasculitis; there are case reports of nasal tip necrosis with non-infectious etiology such as in giant cell arteritis.^3^ To date, no link between VZV and giant cell arteritis has been demonstrated.^4^

VZV infection in patients can lead to serious ophthalmologic complications, such as uveitis, keratitis and blindness. An important exam finding to look for is the Hutchinson sign, which refers to a rash present on the nasal tip in patients with VZV. It indicates the involvement of the nasociliary nerve, a branch of the ophthalmic nerve innervating the eyelid, nose, and eye. The presence of Hutchinson sign indicates a 3.4-fold increase in the risk of ocular inflammation development and a 4-fold increase in the risk of corneal denervation.^5^ Because Hutchinson sign is a strong predictor of serious ocular complications, urgent consultation with ophthalmology is recommended.

Sebaceous gland necrosis has previously been reported with VZVs.^3^ The rapid onset nasal necrosis in the setting of VZV is unclear, but one possibility is the high density of sebaceous glands on nose could predispose rapid necrosis. A history of seborrheic dermatitis and sebaceous hyperplasia in patient 2 may have been contributing factors. The presence of associated demodex mites in biopsies of patient 1 also raises the possibility that demodex associated inflammation could result in an exuberant inflammatory response to VZV, and more rapid epidermal damage and necrosis.

These cases highlight acute onset progressive nontender nasal tip necrosis as an unusual VZV presentation that may occur in isolation or as disseminated disease. Clinicians should consider the possibility of VZV in adult patients, whether immunocompromised or immunocompetent, presenting with nasal tip necrosis to facilitate early detection and treatment.

## Conflicts of interest

None disclosed.
